# Africa Rising, a Narrative for Life Expectancy Gains? Evidence from a Health Production Function

**DOI:** 10.5334/aogh.2307

**Published:** 2019-04-29

**Authors:** Donald Salami, Ahmed Nabil Shaaban, Maria do Rosário Oliveira Martins

**Affiliations:** 1 Global Public Health, Institute of Hygiene and Tropical Medicine, Nova University of Lisbon, PT

## Abstract

**Background:**

The narrative of Africa Rising has increasingly been called into scrutiny, not just as a debate for economic growth and development, but also as a possible link to the surge in life expectancy on the continent. Theoretically, an increase in economic development tends to result in an increase in public health spending and subsequent better health outcomes.

**Objective:**

This paper examines the contribution of economic development and other social determinants to the health status of the African continent and to provide evidence on whether the increase in life expectancy of the past two decades can be largely attributed to the Africa Rising narrative.

**Methods:**

We estimated an empirical health production function, with life expectancy gains as the output of the health care system, and various socio-economic, environmental and lifestyle factors as contributory factors. We fitted a generalized least squares model, using panel data from 52 African countries for the period 1995–2014.

**Findings:**

The estimation shows that while increases in health care spending contributed to life expectancy gains, urbanization rates and improved water access were the major drivers of life expectancy gains with substantially larger impacts in the past two decades.

**Conclusions:**

Overall, the results provide an evidence base for iterating the need to prioritize increasing funding and examine more critically how to improve the efficiency of health spending. It also illustrates potential gains that can be achieved from an inclusive health policy agenda with a broader range of social and economic development issues.

## Introduction

The surge in life expectancy in the African region since 2000 is evidently the greatest since the reverse gains in the 1990s. Life expectancy at birth increased in the region by 9.4 years to 60 years, with some countries experiencing as high as 42% rise between 2000 and 2014 [1]. These remarkable gains have taken place mainly in the context of increased access to antiretrovirals therapy, progress in malaria control and improvements in child survival over time [2]. Over the same period there was a significant economic growth and rise in income across the continent, popularly referred to as ‘Africa Rising’. This rapid economic growth suggests more money for governments to spend on healthcare [3]. However, there is scarce evidence to support that the economic growth has led to higher health spending and higher health spending resulting in better health outcome for the continent. Few studies have been devoted to exploring the contribution of health inputs, particularly health expenditures, as a determinant of health outcomes in Africa.

To assess this relationship, we adopted the health production function framework using aggregate macro data. The theoretical tenets of this approach views health as an output of a health care system that is produced by a set of inputs to the system [4,5]. A relatively large number of empirical studies have adopted this approach as a way of disentangling the overall impact of the health system on the health status of the population from the relative contribution of other broader socioeconomic factors [5,6,7]. The information gained from an aggregate health production function helps annotate the gains from increased health care spending and indicates whether investment in alternative health and socioeconomic agendas may have a better return on health outcomes [5]. This is a central question facing health policy makers in Africa in the context of whether the Abuja Declaration call to increase health expenditure yielded sufficient and commensurate value in health outcomes [8]. Furthermore, this is consequential to achieving universal health coverage and financial risk protection on the continent [9].

The aim of this paper is to examine the relationship between health care expenditure and life expectancy gains as a health outcome in African countries over the period of 1995–2014 by estimating the relative contributions of health care expenditure and the broader socioeconomic determinants of health as inputs of an empirical health production function. Few studies have previously estimated an aggregate health production function for the Sub-Saharan Africa region [10,11]. We seek to build upon these previous studies by incorporating all countries on the continent and by refining the econometric methodology.

## Methods

### Data and Variables

Data used for our analysis comes mainly from the World Bank open database [12]; however, some variables were sourced from other officially recognized international sources. Utilizing a longitudinal study approach, we estimate life expectancy gains between 1995 to 2014 in 52 African countries, as the dependent variable in a health production function. Explanatory variables include factors representing health systems, income, socioeconomic, lifestyle, and environment. These variables reflect key determinants of health appropriate to the circumstances of the African continent and are consistent with previous empirical health production function models. The definition and sources of all variables are listed in Table II of the appendix.

#### Life expectancy (LE)

Life expectancy (LE) at birth is widely used as a proxy measure of population health statue at the aggregate level. A summary indicator of mortality conditions, it represents a proxy of health outcomes and distribution at different demographic, geographic, and economic groups in the population. The dependent variable for our health production function is life expectancy (LE) at birth for the total population. It indicates the average number of years that a newborn is expected to live if current mortality rates continue to apply [13].

#### Health Systems Factors (HF)

Total health expenditure and out-of-pocket spending are included to represent health systems inputs. The specification of the health care resources per capita, in empirical analysis, vary slightly from study to study. We included the monetary measurement represented by the total health expenditure per capita, the sum of public and private health spending as a ratio of total population. It constitutes the provision of health services, family planning activities, nutrition activities, and emergency aid designated for health, exclusive of the provision of water and sanitation [12]. The utilization of total health spending will provide a synthesis understanding of the available health funding with the system. Our a priori expectation is a positive association between health care spending and life expectancy gains, under the assumption that increase resources imply an improvement in the level and/or quality of health care. We do not anticipate diminishing returns given the generally low levels of expenditure in Africa in comparison to other, richer regions.

The second contributory variable to the health system included in our model is the out-of-pocket payments measured as a percent of total expenditure on health. Out-of-pocket payments are defined as expenditures borne directly by individuals or private households to health care providers at the time of service use, where neither compulsory nor voluntary insurance covers the full cost [12]. Some health production function studies include out-of-pocket health spending as a main explanatory variable, however, in recent times, it has become a key proxy for access to care and financial protection under the overall framework of the universal health coverage [14,15]. The implicit theory is high out-of-pocket payment is negatively associated with financial protection, causing individuals to incur catastrophic expenditures, and substantially reduces the health-seeking behaviors of households. The reliance on out-of-pocket payments differs considerably amongst African countries, in some countries out-of-pocket spending are as high as 50% or more of current health spending [9], therefore the relative contribution to the health status cannot be predicted a priori.

#### Socioeconomic Factors (SF)

Socioeconomic influences on life expectancy are represented by three variables: income per capita, education, and food availability. Income per capita as measured by GDP per capita represents the level of economic development and wealth of a society. Income has been largely linked to being a major determinant of health status, though a seemingly ambiguous causal link. For the most part, empirical studies have suggested a positive relationship between income levels and health outcomes [7,16]. Higher incomes facilitate access to goods & services (e.g., food, housing, transportation), better working conditions and better living standard, which in turn contributes to improved health and longevity. Converse arguments state, beyond some threshold level of affluence, increasing income may no longer improve health and rather result in adverse risky behaviors and unhealthy lifestyle (e.g., eating habit, alcohol & tobacco consumption), that may negatively affect health [15,17]. Irrespective of the ambiguity of the impact of income on health, our a priori expectation is higher income contributes to improving health, due to low average income per capita across the region [10].

The second socioeconomic variable is educational attainment, measured as the average years of schooling of the population aged 15 and over. It has been widely theorized by researchers that higher education is an investment in better health [18]. Better educated people are typically more knowledgeable about the risks and benefits of different behaviors and better positioned to understand and act accordingly [19,20]. They are also more informed about the availability of health services resulting in greater use of services [7]. Furthermore, education may be associated with improved self-management and subsequent efficiency of medical treatment, particularly for chronic diseases [21]. Therefore, we expect higher education levels to be positively associated with life expectancy gains.

Food availability is the third socioeconomic factor, measured as the gross food production per capita. Food production is based on the sum of price-weighted quantities of different agricultural commodities, covering food crops that are considered edible and that contain nutrients (exclusive of coffee and tea as they have no nutritive value) [12]. The production index is used as a proxy for food availability and food security, which is closely interconnected with nutrition security and subsequently health [22]. When considered at per capita level, the effect of production on health can be validated in relation to warding off famine and prevention of chronic undernutrition or malnutrition. Africa over the years had recorded the proven successes in agricultural development [23], however, the effect on health cannot be predicted a priori, because maintaining food self-sufficiency seems daunting in conflict-affected areas in the continent.

#### Environmental Factors (EF)

Urbanization, carbon dioxide emissions, water access, and sanitation were included as environmental variables. Urban population refers to people living in urban areas as defined by national statistical offices, a percentage of the total population. It is a proxy for a collection of potential negative and positive health-related factors [5]. High degree of urbanization is associated with adverse consequences in congestion, increase in slum settlements, poverty, ill-health and the decline in urban capital per person (e.g., access to services, water, and sanitation, etc.) [24]. However, urbanization is not inherently negative, it can be a positive determinant of health, via the gains in access to better job opportunities, public service/goods, infrastructure, and better health care. The WHO commission on macroeconomics and health affirms that investments in urban health can create major returns on economics and life expectancy of the population [25]. The marginal effect of urbanization will be dependent on the net effect of these two channels, hence the impact on life expectancy is indeterminate.

The second environmental variable is carbon dioxide emissions (metric tons per capita) as a proxy for air pollution. Carbon dioxide emissions are those stemming from the burning of fossil fuels and the manufacture of cement. They include carbon dioxide produced during consumption of solid, liquid, and gas fuels and gas flaring. The negative effect of carbon dioxide (CO_2_) on health and causal link of air pollution mortality have been well documented [26]. We expect to capture the negative effects of air pollution on health outcomes.

The third environmental factor is access to water and sanitation, which tracks two indicators, percentage of the population with access to improved drinking water sources and improved sanitation. Access to an improved water source refers to the percentage of the population using an improved drinking water source. While access to improved sanitation facilities refers to the percentage of the population using improved sanitation facilities. Access to water and sanitation is well documented to reduce the effects of exposure to pollution and disease, minimize contact with harmful contaminants, such as bacteria and viruses, thereby promoting health and wellbeing [27]. Thus, both indicators should have a positive impact on health outcomes.

#### Lifestyle Factors (LF)

Lastly, we included adult alcohol consumption in litres per capita as a lifestyle variable. Total alcohol consumption per capita measured in equivalent litres of pure alcohol (ethanol) consumed per capita per person ages 15+ per year. Excessive alcohol consumption has detrimental health effects. An associated risk factor for numerous chronic diseases (cardiovascular disease, liver cirrhosis, and certain cancers) as well as accidents and violent deaths [28]. A priori expectation is to capture the negative effects of high consumption on health.

#### Econometric Specification

Our basic analytical approach utilizes macro-level panel data to estimate an empirical aggregate health production function. A Cobb-Douglas production function is employed with all variables expressed in logarithmic form. We used panel data of 52 African countries over the period of 1995–2014.

The general form of the health production function was specified as follows:

\ln \;L{E_{i,t}} = {\alpha _i} + {\beta _1}\ln {H_{i,t}} + {\beta _2}\ln {S_{i,t}} + {\beta _3}\ln {E_{i,t}} + {\beta _4}\ln {L_{i,t}} + {e_{i,t}}

with all variables in natural logarithm form **(*ln*)** and *LE_t_* is the life expectancy at birth for country ***i*** in period ***t***; α the country fixed effect to account for country level characteristics that are constant over the period of analysis; and ***e*** is the error term.

H is the vector of health systems factors (which includes – total health expenditure per capita constant US$ PPP, as a measure of health care spending, out-of-pocket expenditure as a percentage of total health expenditure as a proxy for access to care and financial protection).

S is the vector of socioeconomic factors (which includes – GDP per capita constant US$ PPP, as a measure of income, mean years of schooling as a measure of educational attainment, gross food production per capita US$, a proxy for food availability).

E is the vector of environmental factors (urban population as a percentage of the total population as a measure urbanization, CO_2_ emissions metric tons per capita as a proxy measure for air pollution, percentage of the population with access to improved drinking water sources and improved sanitation facilities as a measure of access to water and sanitation).

L is a lifestyle factor represented by alcohol consumption in litres per capita.

### Estimation Method

We fitted a Generalized Least Squares (GLS) model, which was subsequently complemented by an alternative specification to address possible endogeneity issue and lag-effects. The different empirical models are explained below.

Model 1 – Standard model, with all explanatory variables included. GLS model specified with country fixed effects (specified by country dummies), country-specific first-order autocorrelation structures for errors (AR1) and a correction for heteroscedasticity. This specification is as follows:

\ln \;L{E_{i,t}} = {\alpha _i} + {\beta _1}\ln {H_{i,t}} + {\beta _2}\ln {S_{i,t}} + {\beta _3}\ln {E_{i,t}} + {\beta _4}\ln {L_{i,t}} + {e_{i,t}}\;\left[ {Model\ 1} \right]

Model 2: as per model 1, with 5-year lagged explanatory variables. The introduction of lagged variables considers possible delayed effects of key determinants of health on life expectancy. Also corrects for possible endogeneity, due to possible simultaneity between life expectancy and some of the explanatory variables. Choice of the 5 year lag is based on previous literature and maintaining an adequate number of observations for the analysis. Explanatory variables were lagged from 1995 to 2009, to account for delayed effects in assessing life expectancy gains for 2000 to 2014. The specification is as follows:

\ln \;L{E_{i,t}} = {\alpha _i} + {\beta _1}\ln {H_{i,t - 5}} + {\beta _2}\ln {S_{i,t - 5}} + {\beta _3}\ln {E_{i,t - 5}} + {\beta _4}\ln {L_{i,t - 5}} + {e_{i,t}}\left[ {Model\ 2} \right]

All models were fitted using the GLS function of the nlme R package [29], with the default method of maximizing the restricted loglikelihood (REML). Model accuracy and goodness of fit were based on the normalized root mean square error, and the R^2^ statistic between actual and predicted values. All analyses were performed using R version 3.5.0.

## Results

We estimated two panel-data regressions on the life expectancy at birth, for 52 African countries in the period of 1995–2014 on a broad range of explanatory variables (descriptive statistics are listed in Table 1). Data gaps in specific years were addressed utilizing a standard linear interpolation with linear extrapolation outside of the known data range. However, two countries (South Sudan and Somalia) were excluded from our analysis due to significant missing data for most variables and time-period. All variables in the model were log transformed, as such coefficients will be interpreted as elasticities.

**Table 1:** Descriptive statistics of variables.

	Observation	Mean (Range)

*Life expectancy at birth, total (years)*	1034	57.03 (31.96–75.64)
*Health expenditure per capita, PPP (US$)*	1032	202.06 (5.94–1768.68)
*Out-of-pocket health expenditure (% of total health expenditure)*	1032	39.65% (2.00–80.91)
*Gross domestic product, PPP† (US$)*	1021	41.16 (2.72–384.41)
*Mean years of schooling population 25+ years‡*	959	4.46 (0.90–10.30)
*Food production index*	1037	100.67 (55.77–182.51)
*Urban population (% of total)*	1037	39.53% (7.21–86.92)
CO_2_ emissions (metric tons per capita)*	1036	112.90 (1.57–1004.37)
*Improved water source (% of population with access)*	1025	68.72% (19.50–99.90)
*Improved sanitation facilities (% of the population with access)*	1038	37.26% (3.00–98.40)
Total alcohol consumption, per capita*‡	934	305.50 (1.00–1189.00)

^†^ – Divided by 100; * – Multiplied by 100. ^‡^ Total alcohol consumption and mean years of schooling had the most data gaps, with a missing analysis of 10.2% and 7.8% respectively. This was addressed using standard linear interpolation with linear extrapolation outside of the known data range.

Results from our standard model (model 1, Table 2), show that while adjusting for other factors, increase in health expenditure, income per capita, education, urbanization, and access to sanitation over time have a positive and statistically significant impact on life expectancy gains. Surprisingly out of pocket expenditure and carbon dioxide emission had a positive and statistically significant coefficient, which is contrary to a priori expectations. Likewise, improved water access was contrary to a priori expectations with a negative but insignificant coefficient. The strength and significance of all coefficients were notably impacted with the introduction of the lagged variables in the subsequent model. Model 2 is preferred as it considers delayed effects and partially addressed endogeneity issues.

**Table 2:** Regression coefficients of model estimations.

Explanatory Variables	Model 1 – Life expectancy coefficient (t-ratio)	Model 2 – Life expectancy coefficient (t-ratio)

*Health Expenditure*	0.0023 (2.6494)	0.0027 (2.8082)
*Out-of-pocket Expenditure*	0.0015 (1.9770)	0.0009 (0.8500)
*Income*	0.0064 (4.9465)	0.0146 (7.4467)
*Education*	0.0132 (5.4185)	0.0190 (7.7176)
*Food Production*	–0.0007 (–1.9799)	–0.0021 (–2.4424)
*Urbanization*	0.3537 (46.3057)	0.2385 (22.3734)
*CO_2_ emissions*	0.0019 (2.2721)	–0.0002 (–0.2572)
*Water Access*	–0.0156 (–1.7724)	0.1054 (8.1903)
*Sanitation Access*	0.0402 (6.9397)	0.0471 (8.4221)
*Alcohol Consumption*	0.0000 (0.1579)	–0.0003 (–0.7965)
*Intercept*	2.6208 (0.0467)	2.5203 (0.0322)
*Observations (countries)*	1040 (52)	780 (52)
*NRMSE Accuracy*	0.985	0.990
*R*^2^	0.813	0.911

Model 1 = Generalized least square regressions, with country-fixed effects, error terms following a country-specific AR(1) and correction for heteroskedasticity. Model 2 = As Per model 1, with 5-year, lagged explanatory variables. NRMSE = normalized root mean square error. R^2^ = Efron’s pseudo r-squared.

Table 2 shows the coefficients for model 2 estimation, with the following specific findings: the coefficient of urbanization and access to water were positive and statistically significant, suggesting that a 1 percent increase in urbanization rates and access to improved water source increase life expectancy gains by about 0.24% and 0.11% respectively, other variables held at a constant. The coefficient of health expenditure, income per capita, education and sanitation access were positive and statistically significant. However, the practical significance of their impact on life expectancy at a 1 percent change level is considerably low. Their contribution to life expectancy gains over the review period is represented in Table 3.

**Table 3:** Relative contributions of the explanatory variables to life expectancy gains over time: 1995 to 2009 (from estimated coefficients in Table 2).

Explanatory variables	Regression Coefficient^1^	Contribution to Life Expectancy		1995 Value	*2009 Value*

		%	Months		

*Health Expenditure*	0.0027**	0.21	1.4	123.6	264.8
*Out-of-pocket Expenditure*	0.0009	ns	ns	43.9	37.4
*Income*	0.0146**	1.11	7.7	2403.8	5154.9
*Education*	0.0190**	0.51	3.5	3.6	4.7
*Food Production*	–0.0021*	–0.02	–0.13	94.6	103.7
*Urbanization*	0.2385**	3.36	23.1	35.8	41.3
*CO_2_ emissions*	–0.0002	ns	ns	0.9	1.2
*Water Access*	0.1054**	1.56	10.7	62.1	72
*Sanitation Access*	0.0471**	0.67	4.6	33.9	39.1
*Alcohol Consumption*	–0.0003	ns	ns	2.9	3.1
*Countries Average*			687.6	

1 = model 2, with 5-year, lagged explanatory variables. Regression based on 780 Observations across 52 countries. Significant code: ** indicates significance at 1%; * indicates at 5%; ‘ns’ indicates not significant.

The direction and significance of the food production coefficient remain unchanged even after accounting for lagged effects in model 2. Thereby suggesting the variable has a negative impact on life expectancy, a contradiction with a priori expectations.

Lastly, the result in Table 1 indicates that a change in carbon dioxide emission and alcohol consumption may adversely impact life expectancy outcome, though not statistically significantly. However, these results reflect the impact of lag in time before these variables affect an individual’s health and the relative marginal changes in both variables over time in African countries.

Table 3 presents the relative contributions of the explanatory variables to life expectancy gains over time. During the period under review, the most significant contributor was urbanization with a resultant gain of 23.1 months of life expectancy. Another major contributing variable to gains in life expectancy over the two decades was improved water access, then followed by income per capita, and then access to sanitation and education. Health expenditure and income per capita had the most significant growth by 114% respectively from 1995 to 2009, but surprisingly this growth did not yield a commensurate impact on life expectancy gains. These results conclude that the changes in health expenditure over the review period have made a relatively small contribution to the increase in life expectancy in African countries.

## Discussion

Utilizing a macro-perspective health production function approach, we measured the impact of the health care system inputs in conjunction with other contributory factors on life expectancy gains in African countries. The results of our empirical model are consistent with the conclusions of Or [30], which suggested that environmental factors are more important than health systems inputs in explaining variations in life expectancy gains. Our findings are also consistent with other empirical health production function studies: an increase in health expenditure, income, and better education have a favorable impact on life expectancy gains [7,10,15].

Health care expenditure contributed marginally to the gains in life expectancy in African countries over the past two decades. This is rather startling, as health expenditure per capita surged by 114% in real terms from 1995 to 2009 (from USD PPP 123.6 in 1995 to USD PPP 264.8 in 2009 in constant terms) [12]. The theoretical assumption of increased economic development in the region leading to increased health spending and substantially contributing to better health outcomes was not well supported by our results. Likewise, it contradicts the proposition of Nixon et al. [7], stating slight changes in health expenditure of developing countries would almost certainly lead to bigger impacts on health outcomes. This relatively small contribution of health expenditure might be explained by large financing gaps and low domestic investments in relation to the health needs in African regions [31,32]. To assess the sensitivity of our coefficient estimates, in view of endogeneity and collinearity issues, we estimated an alternative specification by excluding GDP per capita. Two major variables that are highly correlated in a health production function is GDP per capita and health expenditure per capita. Theoretically, instrumental variables could be used to address this issue, though in practice it is challenging to identify a plausible instrument and results prove sensitive to the choice of instruments to provide reliable estimates [33]. Specifications excluding GDP per capita imply a larger impact of health expenditure, suggesting that when GDP is omitted income effects that are unrelated to health expenditure are captured. This scenario also assumes that the impacts of GDP are indirect, i.e. through health expenditure. Health expenditure remained statistically significant in this alternative specification (See Table I in the appendix) suggesting the coefficient estimates to be the actual contribution of health expenditure to life expectancy gains.

**Figure 1:**
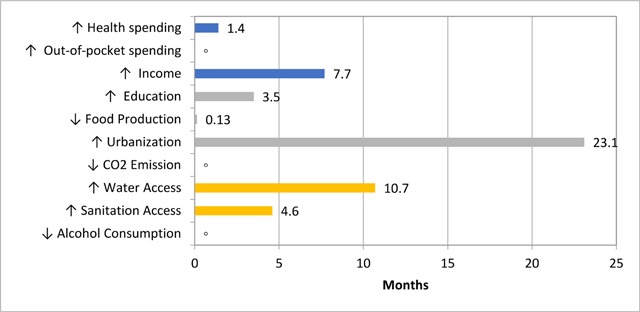
Life expectancy gains associated with percentage change in explanatory variables over time. °indicates not significant.

Urbanization and improved water access are found to be the major contributing factors to gains in life expectancy during the review period. Urbanization rates had a substantial (15.1%) change in real terms from 1995 to 2009, with a resultant gain of 23.1 months of life expectancy (Figure 1). Our a priori expectation for urbanization rates was indeterminate as it could inherently have a positive or negative impact on health outcomes. The positive effects of urbanization on life expectancy, as suggested by our results, are indisputable given the explosive city growth rates since the 1960s. Indeed Africa is now the second-fastest urbanizing continent [34]. This growth can be postulated to have resultant returns on the continent’s economic growth, fostering better income, and better income can be linked to improved health outcomes [35]. However, we do not negate the potential negative impact of high urbanization rates on health outcomes over time, arguably from the stance of it leading to increased pollution and congestion [24]. On a cautionary note, the true marginal effect of urbanization should be considered as the net effect of both channels. Similarly, improved drinking water source had a 16% increase in real terms from 1995 to 2009, with a relative contribution of 13.0 months to life expectancy (Figure 1). These results are consistent with the conclusions of other previous studies assessing the impact of water access on health [36]. Improved water access has been associated with the prevention of a tenth of the global disease burden and prevention of dehydration, which has a direct impact on population health outcomes. Likewise, it is also reasonably linked to improving food security, livelihood choices, poverty reduction and educational opportunities, which indirectly impacts health outcomes in African countries [37]. The gain in improved water access is an imperative prerequisite to achieving most of the dimensions of the sustainable development goals in Africa.

Our findings indicate that an increase in food production, a decrease in carbon dioxide emission and alcohol consumption, may not have contributed to the gains in life expectancy within the period. A probable reason for the negative effect of food production might be due to variations in annual production level for drought-stricken and conflict-affected areas in the continent [38]. To explore this assumption, we estimated an alternative specification of our model, with the addition of a year-specific time dummy variable (details not shown). The direction remained unchanged, but became non-significant suggesting the effects of specific time-period shocks in food production with restrictive impacts on life expectancy. The coefficient for carbon dioxide emission and alcohol consumption became negative and significant with control for specific years. As earlier stated, a plausible explanation for results of carbon dioxide (CO_2_) emission and alcohol consumption is the reflection of a long lag in time before these variables affect a person’s health. Furthermore, it is worth mentioning that the African region is notably still one of the lowest global CO_2_ emitters [39,40,41]. And that the marginal effects of CO_2_ emission on population health are also dependent on the duration of exposure and the balancing oxygen concentration in the atmosphere at a given time [42]. In an equivalent manner, the continent has somewhat of a low alcohol-attributable burden of disease, which is attributed to lag time and likely data paucity [43]. It would be more informative to explore alternative model specifications with longer lags on these variables. We did not pursue this, as longer lags would reduce the number of data observations in our models, which will inadvertently lead to weaker effects.

## Conclusions

Our results not only provide some new evidence on the contributors to life expectancy gains in African countries over the past two decades but raises several important considerations for policymakers. The observed marginal effect of health financing gives an evidence base for iterating the need to prioritize bridging the large gaps in healthcare financing, increase domestic investments in health systems, and examine critically how to improve the efficiency of health spending in African countries as already proposed by the Abuja Declaration. The policy implications of other findings are also important, as they illustrate potential gains that can be achieved from inclusive healthcare agendas with a broader range of social and economic development issues. Our model specifications followed the best methodological practice, however, the limitations of the macro-level approach which demonstrates only aggregate effects and not country-level differences should be considered when interpreting the findings. Our study is broadly consistent with the previous health production function study for Sub-Sahara Africa countries and supports the general opinions of other similar empirical studies.

## Appendix

**Table I:** Alternative model specification excluding GDP per capita.

Explanatory Variables	Full Model^1^	Model excluding Income^2^

*Health Expenditure*	0.0027 (2.8082)	0.0041 (4.6023)
*Out-of-pocket Expenditure*	0.0009 (0.8500)	–0.0000 (–0.0136)
*Education*	0.0190 (7.7176)	0.0281 (10.0765)
*Food Production*	–0.0021 (–2.4424)	–0.0013 (–1.4477)
*Urbanization*	0.2385 (22.3734)	0.2811 (26.5612)
*CO_2_ emissions*	–0.0002 (–0.2572)	0.0011 (1.1730)
*Water Access*	0.1054 (8.1903)	0.0412 (5.2916)
*Sanitation Access*	0.0471 (8.4221)	0.0686 (12.2647)
*Alcohol Consumption*	–0.0003 (–0.7965)	–0.0001 (–0.6496)
*Income*	0.0146 (7.4467)
*Intercept*	2.5203 (0.0322)	2.5710 (0.0293)
*Observations (countries)*	780 (52)	780 (52)
*NRMSE Accuracy*	0.990	0.990
*R^2^*	0.911	0.907

^1^ GLS Model, with country-fixed effects, error terms following a country-specific AR(1) and correction for heteroskedasticity, with 5-year lagged explanatory variables. ^2^ As per full model specification, excluding income (GDP per capita).

**Table II:** Definition and sources of all variables.

Variable	Definition	Aggregation Method	Source

Life expectancy at birth, total (years)	Life expectancy at birth indicates the number of years a newborn infant would live if prevailing patterns of mortality at the time of its birth were to stay the same throughout its life.	Total (years) Weighted average	World Bank
Health expenditure per capita	Total health expenditure is the sum of public and private health expenditures as a ratio of total population. It covers the provision of health services (preventive and curative), family planning activities, nutrition activities, and emergency aid designated for health but does not include the provision of water and sanitation.	PPP (constant 2011 international $) Weighted average	World Bank
Out-of-pocket expenditure	Out of pocket expenditure is any direct outlay by households, including gratuities and in-kind payments, to health practitioners and suppliers of pharmaceuticals, therapeutic appliances, and other goods and services whose primary intent is to contribute to the restoration or enhancement of the health status of individuals or population groups. It is a part of private health expenditure.	% of total expenditure on health Weighted average	World Bank
Gross domestic product – GDP, PPP (constant 2011 international $)	PPP GDP is gross domestic product converted to international dollars using purchasing power parity rates. GDP is the sum of gross value added by all resident producers in the economy plus any product taxes and minus any subsidies not included in the value of the products.	PPP (constant 2011 international $) Weighted average	World Bank
Mean years of schooling population 25+ years	An average number of completed years of education of a country’s population aged 25 years and older, excluding years spent repeating individual grades.	Total (years) Weighted average	UNESCO Institute for Statistics
Food Production index (Production Indices)	Food production index covers food crops that are considered edible and that contain nutrients. Coffee and tea are excluded because, although edible, they have no nutritive value.	Gross per capita Production Index Number (2004–2006 = 100): International US$	Food and Agriculture Organization of the United Nations (FAO)
Urban population (% of total)	Urban population refers to people living in urban areas as defined by national statistical offices.	% of population Weighted average	World Bank
CO_2_ emissions (metric tons per capita)	Carbon dioxide emissions are those stemming from the burning of fossil fuels and the manufacture of cement. They include carbon dioxide produced during consumption of solid, liquid, and gas fuels and gas flaring.	Metric tons per capita	World Bank
Improved water source (% of the population with access)	Access to an improved water source refers to the percentage of the population using an improved drinking water source. The improved drinking water source includes piped water on premises (piped household water connection located inside the user’s dwelling, plot or yard), and other improved drinking water sources (public taps or standpipes, tube wells or boreholes, protected dug wells, protected springs, and rainwater collection).	% of population Weighted average	World Bank
Improved sanitation facilities (% of the population with access)	Access to improved sanitation facilities refers to the percentage of the population using improved sanitation facilities. Improved sanitation facilities are likely to ensure hygienic separation of human excreta from human contact. They include flush/pour flush (to the piped sewer system, septic tank, pit latrine), ventilated improved pit (VIP) latrine, pit latrine with slab, and composting toilet.	% of population Weighted average	World Bank
Total alcohol consumption per capita (liters of pure alcohol, projected estimates, 15+ years of age)	Total alcohol consumption per capita is based on projections for the amount of alcohol consumption (liters of pure alcohol) per person ages 15+ per year.	Liters of alcohol consumed per capita. Weighted average	World bank
